# The role of sports clubs in helping older people to stay active and prevent frailty: a longitudinal mediation analysis

**DOI:** 10.1186/s12966-017-0552-5

**Published:** 2017-07-14

**Authors:** Paul Watts, Elizabeth Webb, Gopalakrishnan Netuveli

**Affiliations:** 1 School of Health, Sport and Bioscience, University of East London, Water Lane, London, E15 4LZ UK; 2 The International Centre for Lifecourse Studies in Society and Health, University College London, Gower Street, London, WC1E 6BT UK; 3 Institute for Health and Human Development, University of East London, Water Lane, London, E15 4LZ UK

**Keywords:** Physical activity, Sports clubs, Frailty, Older adults

## Abstract

**Background:**

Frailty is a common syndrome in older adults characterised by increased vulnerability to adverse health outcomes as a result of decline in functional and physiological measures. Frailty predicts a range of poor health and social outcomes and is associated with increased risk of hospital admission. The health benefits of sport and physical activity and the health risks of inactivity are well known. However, less is known about the role of sports clubs and physical activity in preventing and managing frailty in older adults. The objective of this study is to examine the role of membership of sports clubs in promoting physical activity and reducing levels of frailty in older adults.

**Methods:**

We used data from waves 1 to 7 of the English Longitudinal Study of Ageing (ELSA). Survey items on physical activity were combined to produce a measure of moderate or vigorous physical activity for each wave. Frailty was measured using an index of accumulated deficits. A total of sixty deficits, including symptoms, disabilities and diseases were recorded through self-report and tests. Direct and indirect relationships between sports club membership, levels of physical activity and frailty were examined using a cross-lagged panel model.

**Results:**

We found evidence for an indirect relationship between sports club membership and frailty, mediated by physical activity. This finding was observed when examining time-specific indirect pathways and the total of all indirect pathways across seven waves of survey data (Est = −0.097 [95% CI = −0.124,-0.070], *p* = <0.001).

**Conclusions:**

These analyses provide evidence to suggest that sports clubs may be useful in preventing and managing frailty in older adults, both directly and indirectly through increased physical activity levels. Sports clubs accessible to older people may improve health in this demographic by increasing activity levels and reducing frailty and associated comorbidities. There is a need for investment in these organisations to provide opportunities for older people to achieve the levels of physical activity necessary to prevent health problems associated with inactivity.

**Electronic supplementary material:**

The online version of this article (doi:10.1186/s12966-017-0552-5) contains supplementary material, which is available to authorized users.

## Background

Frailty is a common condition characterised by a decline in function across physical and cognitive systems and a reduced ability to cope with stressors [1]. Frailty has been shown to predict mortality, disability, falls and hospital admissions [2] which can have consequences for quality of life and health, care and welfare systems [3]. The concept of frailty in older adults is particularly important in the context of worldwide increases in life expectancy [4] and the challenges associated with preventing prolonged morbidity in ageing populations.

The most common measure of frailty in published research papers is the Fried frailty phenotype [5]. The Fried phenotype defines frailty as the presence of three or more of: 1) Unintentional weight loss; 2) Weakness (low grip strength); 3) Self-reported exhaustion; 4) Low walking speed; 5) Low physical activity. However, this phenotype is unsuitable for use in studies examining relationships between physical activity and frailty, as physical activity is a component of the frailty phenotype itself. An alternative method of operationalising frailty is using indices of accumulated ‘deficits’ in health [6]. Deficits can be symptoms, signs, diseases, disabilities or clinical measures of health and/or illness. Frailty by accumulated deficits theorises that the more negative outcomes a person experiences, the more likely that person is to be frail [7]. There are 70 deficits in the original frailty index, but these are not a fixed set of variables and indices are estimated to be robust when a minimum of 30 items are considered [6]. In the presence of many deficits, the accumulation of deficits is more important than the specific type of deficits [8]. Measures of frailty by accumulated deficits recognise that frailty is a concept that is multifactorial, complex and dynamic.

A recent review paper [9] concluded that identifying early predictors of frailty is essential for the development of interventions to help prevent and manage frailty in older adults and that this may be achieved through the longitudinal investigation of correlates of frailty. Participation in sports and physical activity may be one such predictor of frailty, but there has been little previous research in this area. Meta-analyses [10] have reported the positive effect of physical activity on individual measures of physical functioning including gait speed, mobility and balance [11]. However, the health benefits of sports participation for older adults are less well evidenced. A longitudinal study of 7456 adults with a mean age of 56 years old at baseline [12] found that regular sports participation was associated with reduced risk of mortality, whilst randomised controlled trials have shown that sports participation can improve cardiovascular health, cognitive functioning and muscle strength [13, 14]. Very little is known about the role of sports club membership in promoting physical activity and improving the health of older adults. A recent cross-sectional study has reported associations between sports club membership and cardiovascular health in adults aged 30–64 years [15], but this remains and under-researched area, especially for older adults.

Many of the health outcomes that have been found to be related to participation in sport and physical activity are components of frailty measures [5–7]. Furthermore, a recent longitudinal study found that regular vigorous physical activity was associated with reduced frailty in adults over 50 years old [16]. Evaluations of interventions have demonstrated that physical activity can be useful in the management of frailty [17], and moreover that physical activity interventions can reverse the progression of frailty [18]. A longitudinal study by Peterson et al., [19] found that, compared to participants who reported doing strengthening exercises and walking for exercise, those who reported only lighter activities (e.g. housework, walking to the shops or volunteer work) had almost three times the odds of frailty at 5 years follow-up. Cesari et al., [20] found that, in a randomised controlled trial of the effectiveness of strengthening exercises on frailty, the intervention group had a 10% reduction in frailty prevalence at 12 months follow-up relative to a control group that received an educational intervention.

It is recommended that older adults participate in moderate and/or vigorous physical activity totalling 150 min per week, plus strengthening exercises for major muscle groups on two or more days [21]. However, just 51% of men and 42% of women aged 65 to 74 in England met these guidelines in 2012 [22] and data from Sport England suggest that at 26%, participation in sporting activities at least once per week is lowest among adults aged over 55 [23]. Little is known about whether infrastructure designed to support participation in sport and physical activity influences older adults’ likelihood of meeting recommended levels of physical activity, but membership of a sports club may contribute through opportunities for moderate and vigorous physical activity and for strengthening exercises. Analyses of Sport England’s ‘Active People Survey’ [24] showed that membership of a sports club is associated with achieving the recommended physical activity levels. Furthermore, sports clubs are an abundant resource in England: 99% of adults live within twenty minutes travel of a sports facility [25]. Access to sports infrastructure is an important determinant of older adult’s participation [25], but fewer than 20% of adults aged 55 years and older are a member of a sports club [23]. Sports clubs may therefore be a valuable and under-used resource for promoting physical activity and reducing frailty in older adults.

Older adults are a population subgroup that may benefit most from increased participation in sport, but little is known about whether sports club membership helps older adults to improve or maintain physical activity levels and prevent frailty. Therefore, in this paper, we have sought to address the following research questions:

- Is membership of a sports club associated with changes in frailty over time?
- Is the relationship between membership of a sports club and changes in frailty mediated by physical activity?

## Methods

### English Longitudinal Study of Ageing (ELSA)

We used ELSA data [26] on demographic, socioeconomic, behavioural and health-related characteristics collected by household survey every two years. ELSA participants are adults aged over 50 years old, originally recruited from previous Health Survey for England participants. The first wave of data collection was in 2002 and repeated measurements on participants have been taken by household survey every two years, up to and including and wave 7, which was completed in 2015. ELSA participants complete face to face surveys using computer-assisted interviewing and self-completion surveys with the support of trained interviewers. Further details on the methods used to collect ELSA data have been published previously [26].

### Sports club and physical activity measures

Each wave of ELSA has three questions on physical activity participation as follows: 1) Do you take part in sports or activities: 1) …that are vigorous; 2) …that are moderately energetic; 3) …that are mildly energetic. Each question has the following possible responses: i) more than once a week; ii) once a week; iii) one to three times a month, or; iv) hardly ever, or never. The responses to these questions were reduced to a binary score for each participant at each wave to represent participation in moderate and/or vigorous physical activity (MVPA): 1) No MVPA (no MVPA or MVPA less than once per week); 2) regular MVPA (MVPA at least once per week). This measure of MVPA has excellent convergent validity with many physical, biochemical and psychosocial risk factors [27]. Sports club membership at each survey wave was recorded from a single item in the ELSA household survey: *“Are you a member of any of these organisations, clubs or societies: …Sports clubs, gyms, exercise classes?”*

### Frailty measures

Frailty was operationalised using an index of accumulated deficits including 60 items covering: a) self-reported difficulty with 23 different daily activities; b) self-reported general health and wellbeing; c) self-reported specific health conditions, and; d) cognitive tests. Each item was transformed to a value between 0 and 1 as follows: a) for binary items (e.g. difficulty walking 100 yards) ‘1’ indicates a deficit and ‘0’ indicates no deficit; b) for Likert scale items (e.g. self-reported eyesight) ‘Poor’ = 1; ‘Fair’ = 0.75; Good = 0.5; Very good = 0.25; Excellent = 0; c) continuous variables (e.g. score on a word recall test) were converted into quintiles and recoded as follows: 5th quintile = 1; 4th quintile = 0.75; 3rd quintile = 0.5; 2nd quintile = 0.25; 1st quintile = 0. All items were summed and the total was divided by the number of items present for each respondent, resulting in a frailty score for each respondent ranging between 0 and 1. Frailty scores were calculated for respondents with at least 30 out of 60 non-missing items [6]. This method of creating a frailty index has been used previously with ELSA data and a full list of items included in the index has been reported by Marshall et al. [28] and is also presented in Additional file 1.

### Statistical analyses

All analyses were conducted in Mplus version 7.4 [29]. We limited our analyses to adults over 50 years old with sufficient non-missing items to create frailty indices at wave 1. This resulted in an initial analytic sample of 11,345. Of this initial sample, data were available on: 8808 participants (77.6%) at wave 2; 7562 (67.4%) at wave 3; 6649 (58.6%) at wave 4; 6264 (55.2%) at wave 5; 5681 (50.0%) at wave 6; and, 4765 (42%) at wave 7. Mplus uses the Full Information Maximum Likelihood (FIML) approach for missing data, which enables the use of all available data points, including observations with some missing responses.

**Fig. 1:**
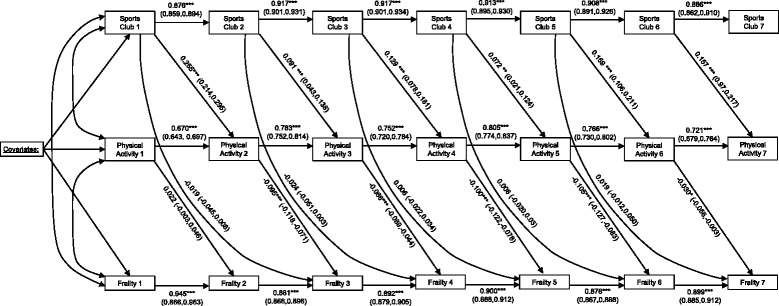
Cross-lagged panel model to assess time-specific direct and indirect ‘effects’*.* Estimates (95% Confidence intervals); *P < 0.05; **P < 0.01; ***P < 0.001

In a mediational model, the associations between the independent variable ‘X’ (sports club membership), the mediating variable ‘M’ (physical activity) and the outcome ‘Y’ (frailty) were examined [30, 31]. A cross-lagged panel model (CLPM) was used to estimate time-specific direct and indirect ‘effects’ (see Fig. 1). The CLPM [32] is composed of two parts: i) an autoregressive part, where the measures of X, M and Y at time *t* are regressed on the same variable measured at the previous time (*t* – 1), and; ii) a cross-lagged part in which M at time *t* is regressed on X at time *t* – 1 (path *a*), Y at time *t* is regressed on M at time *t* – 1 (path *b*), Y at time *t* is regressed on X at time *t* – 2 (path *c*, ‘total effect’), and Y time *t* is regressed on X at time *t* – 2, while controlling for while controlling for M (path *c’*, ‘direct effect’). These path coefficients are used to estimate the time-specific indirect ‘effects’, by calculating the product of path *a* and path *b*. The ‘total indirect effect’ is calculated as the sum of all indirect pathways between sports club membership at wave 1 and frailty at wave 7 [32, 33].

The CLPM (Fig. 1) was adjusted for age, gender and NSSEC social class, highest education qualification, ethnicity, whether currently living with a spouse or partner, smoking status and employment status. Bias-corrected bootstrapped confidence intervals were calculated for all estimates of direct and indirect pathways in the CLPM.

## Results

Descriptive statistics for a total of 11,345 respondents across the seven ELSA survey waves are shown in Table 1. Overall sports club participation was highest at 20.8% in wave 7 and lowest at 16.3% at wave 1. The proportion of respondents reporting MVPA at least once per week was similar across all waves, ranging from 61.3% at wave 2 to 57% at wave 5. The mean frailty scores recorded at each wave were similar, with the lowest mean (0.149) recorded at wave 1 and the highest (0.165) recorded at wave 5. Proportions of frailty across covariates are shown in Table 2 and proportions of missing data are displayed in Additional file 2.

**Table 1:** Descriptive statistics for ELSA survey waves 1 to 7

	Wave 1		Wave 2		Wave 3		Wave 4		Wave 5		Wave 6		Wave 7	
Dependant variables	N	%/SD	N	%/SD	N	%/SD	N	%/SD	N	%/SD	N	%/SD	N	%/SD
Total (%)	11,345	100	8808	100	7562	100	6649	100	6264	100	5681	100	4915	100
Sports club membership (%)	1893	16.69	1496	16.98	1233	16.31	1159	17.43	1144	18.26	1071	18.85	1023	20.81
MVPA at least once per week (%)	6703	59.08	5399	61.30	4546	60.12	3938	59.23	3568	56.96	3293	57.97	2836	57.70
Mean Frailty index (SD)	0.149	0.116	0.157	0.118	0.159	0.118	0.161	0.118	0.165	0.124	0.161	0.129	0.160	0.127
Covariates	N	%	N	%	N	%	N	%	N	%	N	%	N	%
Aged 50 to 59 years (%)	4551	38.63	3307	37.55	2934	38.80	2675	40.23	2670	42.62	2529	44.52	2303	46.86
Aged 60 to 69 years (%)	3377	28.66	2701	30.67	2350	31.08	2142	32.22	2078	33.17	1914	33.69	1721	35.02
Aged 70 to 79 years (%)	2566	21.78	1968	22.34	1639	21.67	1392	20.94	1201	19.17	1019	17.94	769	15.65
Aged over 80 years (%)	1288	10.93	832	9.45	639	8.45	440	6.62	315	5.03	219	3.85	122	2.48
Men (%)	5146	45.36	3947	44.82	3356	44.39	2918	43.89	2743	43.78	2475	43.57	2123	43.20
Women (%)	6198	54.64	4860	55.18	4205	55.61	3730	56.11	3522	56.22	3205	56.43	2791	56.80
NSSEC - Managerial and professional (%)	3360	30.50	2835	32.82	2506	33.74	2273	34.76	2182	35.30	2035	36.26	1804	37.13
NSSEC - Intermediate (%)	2581	23.43	2063	23.89	1798	24.21	1595	24.39	1534	24.82	1368	24.38	1205	24.80
NSSEC - Routine and manual (%)	5077	46.08	3739	43.29	3123	42.05	2672	40.86	2465	39.88	2209	39.36	1849	38.06
Lives with a spouse or partner	7313	65.39	5442	62.35	4446	59.45	3816	57.80	3666	66.35	3266	66.52	2760	65.82
Doesn’t live with a spouse or partner	3871	34.61	3286	37.65	3032	40.55	2786	42.20	1859	33.65	1644	33.48	1433	34.18
White ethnicity	11,019	97.19	8608	97.74	7403	97.91	6507	97.88	6118	97.68	5543	97.59	4799	97.66
Non-white ethnicity	319	2.81	199	2.26	158	2.09	141	2.12	145	2.32	137	2.41	115	2.34
No qualifications	5353	51.71	3838	47.90	3177	46.00	2683	44.16	2428	42.43	2137	41.11	1734	38.49
Intermediate qualifications	1802	17.41	1477	18.43	1305	18.89	1196	19.68	1154	20.17	1070	20.58	987	21.91
Higher education or above	3197	30.88	2697	33.66	2425	35.11	2197	36.16	2140	37.40	1991	38.30	1784	39.60
Smoker	2018	17.79	1139	13.24	1013	13.40	801	12.14	694	11.24	559	9.85	489	10.60
Non-smoker	9324	82.21	7463	86.76	6546	86.60	5797	87.86	5480	88.76	5119	90.15	4125	89.40
Currently in paid employment	4069	35.87	2842	32.27	2242	29.65	1703	25.61	1319	21.06	953	16.78	500	10.17
Not in paid employment	7273	64.11	5965	67.72	5318	70.33	4944	74.36	4943	78.91	4726	83.19	4414	89.81

**Table 2:** Distribution of frailty across covariates and associations between covariates and frailty at wave 1

Covariates	Mean frailty index	Est	95% CI
Aged 50 to 59 years (%)	0.116	Ref	
Aged 60 to 69 years (%)	0.140	0.023***	(0.018, 0.028)
Aged 70 to 79 years (%)	0.173	0.056***	(0.051, 0.062)
Aged over 80 years (%)	0.227	0.111***	(0.104, 0.118)
Men (%)	0.137	Ref	
Women (%)	0.014	0.017***	(0.010, 0.023)
NSSEC - Managerial and professional (%)	0.139	Ref	
NSSEC - Intermediate (%)	0.149	0.014**	(0.005, 0.024)
NSSEC - Routine and manual (%)	0.210	0.038***	(0.029, 0.046)
Doesn’t live with a spouse or partner	0.129	Ref	
Lives with a spouse or partner	0.183	−0.028***	(−0.035, −0.022)
White ethnicity	0.148	Ref	
Non-white ethnicity	0.174	0.050***	(0.032, 0.069)
No qualifications	0.155	Ref	
Intermediate qualifications	0.144	−0.035***	(−0.044, −0.026)
Higher education or above	0.121	−0.048***	(−0.060, −0.037)
Non-smoker	0.146	Ref	
Smoker	0.163	0.030***	(0.023, 0.038)
Not in paid employment	0.181	Ref	
Currently in paid employment	0.091	−0.088***	(−0.095, −0.080)

***p < 0.001, **p < 0.01 and *p < 0.05

The CLPM had good model fit (RMSEA = 0.039 [90% CI = 0.038, 0.040]; CFI = 0.905; TLI = 0.912). We found evidence for a total indirect effect (sum of all indirect pathways) of sports clubs on frailty, mediated by physical activity (Est = −0.097, [95% CI = −0.124,-0.070], *p* = <0.001). Estimates for all autoregressive paths of sports club membership, physical activity and frailty indicated positive associations between measures of each construct at time *t* and time *t* + 1. Sports club membership at waves 1 to 6 was positively associated with physical activity at time *t* + 1 (time-specific path *a*). Physical activity measured at waves 2 to 6 was negatively associated with frailty at time *t* + 1 (time-specific path *b*). Physical activity at wave 1 was not associated with frailty at wave 2. In all five time-specific indirect pathways examined, there was evidence of indirect associations between sports club membership at time *t* and frailty at time (*t* + 2), mediated by physical activity at time *t* + 1 (see Table 3). There was no evidence of time-specific direct associations between sports club membership and frailty in the CLPM adjusted for MVPA (see Table 3).

**Table 3:** Time-specific direct, indirect and total ‘effects’ for the CLPM

Time-specific pathway	Sports club 1 ➔ Frailty 3		Sports club 2 ➔ Frailty 4		Sports club 3 ➔ Frailty 5		Sports club 4 ➔ Frailty 6		Sports club 5 ➔ Frailty 7	
	Est	95% CI	Est	95% CI	Est	95% CI	Est	95% CI	Est	95% CI
Path *a* (x➔m)	0.255***	(0.214, 0.295)	0.091***	(0.043, 0.138)	0.129***	(0.078, 0.181)	0.072**	(0.021, 0.124)	0.159***	(0.106, 0.211)
Path *b* (m➔y)	−0.022	(−0.003, 0.046)	−0.095***	(−0.118, −0.071)	−0.066***	(−0.089, −0.044)	−0.105***	(−0.127, −0.083)	−0.030*	(−0.056, −0.003)
a*b (indirect ‘effect’)	−0.024***	(−0.032,−0.016)	−0.006**	(−0.010, −0.002)	−0.013***	(−0.019, −0.007)	−0.008*	(−0.013, −0.002)	−0.005*	(−0.009, −0.000)
Path c’ (direct ‘effect’)	−0.019	(−0.045, 0.008)	−0.024	(−0.051, 0.003)	0.006	(−0.022, 0.034)	0.008	(−0.020, 0.036)	0.019	(−0.012, 0.050)
Path *c* (total ‘effect’)	−0.043**	(−0.067,−0.018)	−0.030*	(−0.057, 0.004)	−0.007	(−0.033, 0.020)	0.000	(−0.027, 0.028)	0.014	(−0.015, 0.044)

*CI* Confidence interval

***p < 0.001, **p < 0.01 and *p < 0.05

All covariates in the model were significantly associated with frailty scores (see Table 2). Females were more likely to report higher frailty scores (Est = 0.017 [95% CI = 0.010,0.023]) and increasing age was associated with higher frailty scores (Est = 0.008 [95% CI = 0.004,0.011]). Higher levels of NSSEC social class and higher levels of education were associated with lower frailty scores. White ethnicity, being in paid employment and being a non-smoker was associated with lower frailty scores.

## Discussion

We have observed a relationship between being a member of a sports club and reduced frailty, which is mediated by increases in physical activity associated with sports club membership. These findings suggest that sports clubs may play an important role in helping adults to stay active and to prevent frailty through the increased levels of physical activity amongst sports club members.

In all analyses there was a consistent positive association between sports club membership and physical activity at time-specific intervals. Similarly, physical activity was consistently associated with reduced frailty measured by accumulated deficits. The only exception to this observation was the lack of an association between physical activity measured at wave 1 and frailty measured at wave 2. The indirect relationship between sports club membership and frailty, mediated by physical activity was observed when examining five time-specific indirect pathways and the total of these indirect pathways.

The advantage of the CLPM is that the temporal precedence of sports club membership before physical activity and physical activity before frailty is explicitly modelled at time specific intervals [32, 33]. However, a difficulty is the identification of the optimal time-lag between measurements of these variables [31]. Our analyses have been limited to the time-lag between ELSA survey waves (approximately two years) which is unlikely be the optimal amount of time elapsed to capture the effect of sports club membership on physical activity and subsequently frailty. The timing of the ELSA data collection prevents us from making inferences about the amount of time that is required for membership of member of a sports club, to influence frailty, through increased physical activity.

To allow the examination of continuous inter- and intra-individual trajectories in measures across multiple time points, we also examined a Latent Growth Curve Modelling (LGCM) approach and have presented these methods and results in Additional file 3. The results from the LGCM also showed an indirect relationship between sports club membership and frailty, mediated by physical activity. However, in a mediational framework this LGCM is correlational as the change in sports club membership, physical activity and frailty are depicted concurrently without evidence of temporal precedence [31].

Strengths of this study include the large sample which is representative of the population of people aged 50 years and older living in the community in England. Limitations of the data used in this study include the self-reported measure of physical activity. Previous research has shown than when using self-reported measures of physical activity, older adults are more likely to over-report physical activity levels than younger adults [34]. The measure of physical activity used in our study has been shown to predict various health outcomes [27], and has been shown to be moderately correlated with accelerometer derived physical activity in a sub-sample of ELSA participants [35]. However, little is known about the reliability of this measure. Furthermore, as a binary derived variable it is not possible to examine the influence of physical activities at various frequencies, durations and intensities. This may be important in this study as it is common in physical activity research for more vigorous activities to be recalled more accurately than moderate or light intensity activities [36, 37]. Similarly, the measure of sports club membership lacks specificity. Defined as “*Sports clubs, gyms, exercise classes”,* this single measure does not allow us to investigate which specific types of sports clubs, facilities, activities and resources are the most important contributors to increased activity and reduced frailty.

The levels of data missing in this study due to attrition are common in longitudinal studies [38] and have been reported in previous studies using ELSA data [16, 39–41]. The FIML approach to missing data has been shown to be less biased than other approaches such as pairwise or list wise deletion. However, despite this approach we cannot rule out the risk of bias due to missing data. A further limitation is that we cannot rule out the possibility that there are additional confounding factors that are not accounted for in our models.

Sports clubs may provide opportunities for the types of physical activity most likely to prevent frailty. For example, strengthening exercises have been shown to be more effective in the prevention of frailty than everyday activities such as housework or walking for transportation [19] and aerobic, resistance and flexibility training interventions are effective in reducing frailty [17]. These types of physical activity are likely to require support through access to specialist equipment and facilities and guidance or support from physical activity specialists. Sports clubs are therefore likely to be best equipped to provide opportunities to access the support and resources required to help older adults increase and maintain physical activity levels. Furthermore, sports clubs may represent an opportunity for social interaction (for example through group activities or interaction with staff members) and therefore may be related to cognitive aspects of frailty indices.

## Conclusions

Local authorities in England have a duty to promote healthy lifestyles but are not obliged to provide sport and physical activity infrastructure for their residents. Providing sports, activities and resources that consider the needs of older adults is a challenge for local authority clubs that have experienced substantial cuts to their budgets since 2010. Those designing interventions to prevent frailty through increased participation in sport and physical activity should consider motivators and barriers to sports club membership and physical activity in older adults [42]. Furthermore, an understanding of older adults’ predisposition to participate in sports and physical activity will be important in the design of interventions to prevent frailty through sports club membership and physical activity. Life-course theories suggest that accumulated experiences across the lifespan are useful in understanding physical activity levels in later life [43]. Important transitional experiences such as retirement or life experiences such as births and deaths of loved ones may be important determinants of physical activity trajectories and sports club membership. Similarly, early experiences with sport and physical activity are likely to predict participation in later life and influence the effectiveness of sport and physical activity interventions designed to prevent frailty. Personality characteristics have been shown to be associated with risk of frailty and participation in sport and physical activity [34]. Individual differences in personality characteristics should therefore be considered in the design of interventions.

Future research should use more detailed measures of sports club membership (e.g. types of sports clubs and types of membership) to determine those most likely to encourage participation in heath enhancing physical activity. Similarly, there is a need for studies that include objective measures of the type, frequency, duration and intensity of physical activity that will most effectively prevent frailty.

Experimental studies are needed provide stronger causal evidence of the pathway between sports club membership, physical activity and frailty. Previous studies have demonstrated that recruitment of older adults into exercise classes may be an effective method for improving health through increased physical activity [44]. And, there are examples of effective interventions utilising professional sports clubs as a setting for the delivery of physical activity interventions [45]. However, there is a need for experimental research into the effectiveness of sports club membership for promoting physical activity and reducing frailty. For example, studies that randomise participants to free sports club membership or usual care. Sports clubs are an existing resource that may represent a cost-effective opportunity for older adults to participate in health enhancing physical activity. Therefore, trials to investigate the effectiveness of sports club memberships as a method for increasing physical activity and reducing frailty in older adults are a research priority.

## Additional files

Additional file 1: Full list of deficits included in the accumulated deficits index (adapted from Marshall et al. [28]. (DOCX 15 kb)

Additional file 2: Proportions of missing data. (DOCX 18 kb)

Additional file 3: Latent growth curve model (LGCM). (DOCX 118 kb)

## Abbreviations

CLPM: Cross Lagged Panel Model
ELSA: English Longitudinal Study of Ageing
LGCM: Latent Growth Curve Model
MVPA: Moderate or vigorous physical activity
