# Supplementary material for: The role of sports clubs in helping older people to stay active and prevent frailty: a longitudinal mediation analysis
Source: Int J Behav Nutr Phys Act. 2017 Jul 14;14:95. doi: 10.1186/s12966-017-0552-5 (PMC5512788; doi:10.1186/s12966-017-0552-5)
Supplement: Supplementary file 1 — Full list of deficits included in the accumulated deficits index (adapted from Marshall et al. [28]. (DOCX 15 kb) [file 12966_2017_552_MOESM1_ESM.docx]

**Additional file 1. Full list of deficits included in the accumulated deficits index (adapted from Marshall et al. [28]**

| **Item No** | **Description** | **Variable type** |
| --- | --- | --- |
| 1 | Difficulty with walking 100 yards | Binary |
| 2 | Difficulty sitting for about two hours | Binary |
| 3 | Difficulty getting up from a chair after sitting for long periods | Binary |
| 4 | Difficulty climbing several flights of stairs without resting | Binary |
| 5 | Difficulty climbing one flight of stairs without resting | Binary |
| 6 | Difficulty stooping, kneeling, or crouching | Binary |
| 7 | Difficulty reaching or extending arms above shoulder level | Binary |
| 8 | Difficulty pulling or pushing large objects like a living room chair | Binary |
| 9 | Difficulty lifting or carrying weights over 10 pounds, like a heavy bag | Binary |
| 10 | Difficulty picking up a 5p coin from a table | Binary |
| 11 | Difficulty dressing, including putting on shoes and socks | Binary |
| 12 | Difficulty walking across a room | Binary |
| 13 | Difficulty bathing or showering | Binary |
| 14 | Difficulty eating, such as cutting up your food | Binary |
| 15 | Difficulty getting in or out of bed | Binary |
| 16 | Difficulty using the toilet, including getting up or down | Binary |
| 17 | Difficulty using a map to figure out how to get around in a strange place | Binary |
| 18 | Difficulty preparing a hot meal | Binary |
| 19 | Difficulty shopping for groceries | Binary |
| 20 | Difficulty making telephone calls | Binary |
| 21 | Difficulty taking medications | Binary |
| 22 | Difficulty managing money, (e.g paying bills and keeping track of expenses) | Binary |
| 23 | Difficulty doing work around the house or garden | Binary |
| 24 | Self-reported general health | Likert |
| 25 | Whether respondent has felt depressed much of the time during past week | Binary |
| 26 | Whether respondent felt everything they did during the past week was an effort | Binary |
| 27 | Whether respondent felt their sleep was restless during the past week | Binary |
| 28 | Whether respondent was happy much of the time during the past week | Binary |
| 29 | Whether respondent felt lonely much of the time during the past week | Binary |
| 30 | Whether respondent enjoyed life much of the time during the past week | Binary |
| 31 | Whether respondent felt sad much of the time during the past week | Binary |
| 32 | Whether respondent could not get going much of the time during the past week | Binary |
| 33 | High blood pressure or hypertension (self-reported) | Binary |
| 34 | Angina (self-reported) | Binary |
| 35 | Heart attack (including myocardial infarction or coronary thrombosis) (self-reported) | Binary |
| 36 | Congestive heart failure (self-reported) | Binary |
| 37 | An abnormal heart rhythm (self-reported) | Binary |
| 38 | Diabetes or high blood sugar (self-reported) | Binary |
| 39 | A stroke (cerebral vascular disease) (self-reported) | Binary |
| 40 | Chronic lung disease such as chronic bronchitis or emphysema (self-reported) | Binary |
| 41 | Asthma(self-reported) | Binary |
| 42 | Arthritis (including osteoarthritis , or rheumatism) (self-reported) | Binary |
| 43 | Osteoporosis, sometimes called thin or brittle bones (self-reported) | Binary |
| 44 | Cancer or a malignant tumour (excluding minor skin cancers) (self-reported) | Binary |
| 45 | Parkinson's disease (self-reported) | Binary |
| 46 | Any emotional, nervous or psychiatric problems (self-reported) | Binary |
| 47 | Alzheimer's disease (self-reported) | Binary |
| 48 | Dementia, or any other serious memory impairment (self-reported) | Binary |
| 49 | Self-reported eyesight (while using lenses, if appropriate) | Likert |
| 50 | Self-reported hearing (while using hearing aid if appropriate) | Likert |
| 51 | Whether respondent has fallen down at all /last year /last 2years | Binary |
| 52 | Whether respondent has fractured hip ever /in last 2 years | Binary |
| 53 | Whether respondent has had joint replacement | Binary |
| 54 | Whether respondent has had pain whilst walking | Binary |
| 55 | Identify today's date: day of month | Binary |
| 56 | Identify today's date: month | Binary |
| 57 | Identify today's date: year | Binary |
| 58 | Identify the day of the week? | Binary |
| 59 | Immediate word recall (sample organised into quartiles) | Quartiles |
| 60 | Delayed word recall (sample organised into quartiles) | Quartiles |
