# Supplementary material for: The role of sports clubs in helping older people to stay active and prevent frailty: a longitudinal mediation analysis
Source: Int J Behav Nutr Phys Act. 2017 Jul 14;14:95. doi: 10.1186/s12966-017-0552-5 (PMC5512788; doi:10.1186/s12966-017-0552-5)
Supplement: Supplementary file 2 — Proportions of missing data. (DOCX 18 kb) [file 12966_2017_552_MOESM2_ESM.docx]

**Additional file 2. Proportions of missing data**

|  | **Wave 1** | | **Wave 2** | | **Wave 3** | | **Wave 4** | | **Wave 5** | | **Wave 6** | | **Wave 7** | |
| --- | --- | --- | --- | --- | --- | --- | --- | --- | --- | --- | --- | --- | --- | --- |
| **Covariates** | **N** | **%** | **N** | **%** | **N** | **%** | **N** | **%** | **N** | **%** | **N** | **%** | **N** | **%** |
|  | **Whole wave** | | | | | | | | | | | | | |
| **Total (%)** | 11345 | 100 | 8808 | 100 | 7562 | 100 | 6649 | 100 | 6264 | 100 | 5681 | 100 | 4915 | 100 |
| **Whole wave missing (attrition)** | 0 | 0 | 2537 | 22.36 | 3783 | 33.35 | 4696 | 41.39 | 5081 | 44.79 | 5664 | 49.93 | 6430 | 56.68 |
| **Dependant variables** | **Within wave** | | | | | | | | | | | | | |
| **Sports club membership** | 1406 | 12.39 | 1533 | 17.40 | 1424 | 18.83 | 1282 | 19.28 | 1029 | 16.43 | 1021 | 17.97 | 920 | 18.72 |
| **Physical Activity** | 91 | 0.80 | 101 | 1.15 | 30 | 0.40 | 18 | 0.27 | 8 | 0.13 | 6 | 0.11 | 3 | 0.06 |
| **Frailty index (≤30 non-missing)** | 1 | 0.01 | 1 | 0.01 | 1 | 0.01 | 2 | 0.03 | 1 | 0.02 | 1 | 0.02 | 1 | 0.02 |
| **Covariates** | **N** | **%** | **N** | **%** | **N** | **%** | **N** | **%** | **N** | **%** | **N** | **%** | **N** | **%** |
| **Age** | 0 | 0.00 | 0 | 0.00 | 0 | 0.00 | 0 | 0.00 | 0 | 0.00 | 0 | 0.00 | 0 | 0.00 |
| **Sex** | 1 | 0.01 | 1 | 0.01 | 1 | 0.01 | 1 | 0.02 | 1 | 0.02 | 1 | 0.02 | 1 | 0.02 |
| **NSSEC social class** | 327 | 2.88 | 171 | 1.94 | 135 | 1.79 | 109 | 1.64 | 83 | 1.33 | 69 | 1.21 | 57 | 1.16 |
| **Whether living with spouse or partner** | 161 | 1.42 | 80 | 0.91 | 84 | 1.11 | 47 | 0.71 | 739 | 11.80 | 771 | 13.57 | 722 | 14.69 |
| **Ethnicity** | 7 | 0.06 | 1 | 0.01 | 1 | 0.01 | 1 | 0.02 | 1 | 0.02 | 1 | 0.02 | 1 | 0.02 |
| **Highest qualification** | 993 | 8.75 | 769 | 8.73 | 655 | 8.66 | 573 | 8.62 | 542 | 8.65 | 483 | 8.50 | 410 | 8.34 |
| **Smoking status** | 3 | 0.03 | 6 | 0.07 | 3 | 0.04 | 51 | 0.77 | 90 | 1.44 | 3 | 0.05 | 85 | 1.73 |
| **Employment status** | 3 | 0.03 | 1 | 0.01 | 2 | 0.03 | 2 | 0.03 | 2 | 0.03 | 2 | 0.04 | 1 | 0.02 |
