# Supplementary material for: The role of sports clubs in helping older people to stay active and prevent frailty: a longitudinal mediation analysis
Source: Int J Behav Nutr Phys Act. 2017 Jul 14;14:95. doi: 10.1186/s12966-017-0552-5 (PMC5512788; doi:10.1186/s12966-017-0552-5)
Supplement: Supplementary file 3 — Latent growth curve model (LGCM). (DOCX 118 kb) [file 12966_2017_552_MOESM3_ESM.docx]

**Additional file 3. Latent growth curve model (LGCM)**

*Statistical Analyses*

Latent growth curve models (LGCMs) for seven ELSA waves of sports club membership, MVPA, and frailty were used to examine direct and indirect associations between sports club membership and frailty. LGCMs are appropriate for examining individual developmental trajectories in physical activity and frailty and differences between individuals with repeated measures over time [1]. Furthermore, latent growth curve modelling of sports clubs allows us to model an individual’s propensity to always or never be a member, or to move in and out of sports club membership over the 7 survey waves. LGCMs are characterised by a latent intercept factor to represent the starting point of the growth trajectory and a latent slope to represent the change in the growth curve over time [1].

In a mediational LGCM, the associations between the independent variable ‘X’ (sports club membership), the mediating variable ‘M’ (physical activity) and the outcome ‘Y’ (frailty) were examined [1, 2]. Specifically, the analysis examined: i) whether the slope growth factor of X is associated with the slope growth factor of Y (path *c* or ‘total effect’); ii) whether the slope growth factor of X is associated with the slope growth factor of M (path *a,* Figure A); iii) whether the slope growth factor of M is associated with the slope growth factor of Y, while controlling for the slope growth factor of X (path *b,* Figure A); iv) the product of path *a* and path *b* (the ‘indirect effect’), and; v) whether the slope growth factor of X is associated with the slope growth factor of Y, while controlling for the slope growth factor of M (path *c*’ or the ‘direct effect’, see Figure A). The LCGM was adjusted for age, gender and NSSEC social class, highest education qualification, ethnicity, whether currently living with a spouse or partner, smoking status and employment status.

**Figure A. Mediational Latent Growth Curve Model.**


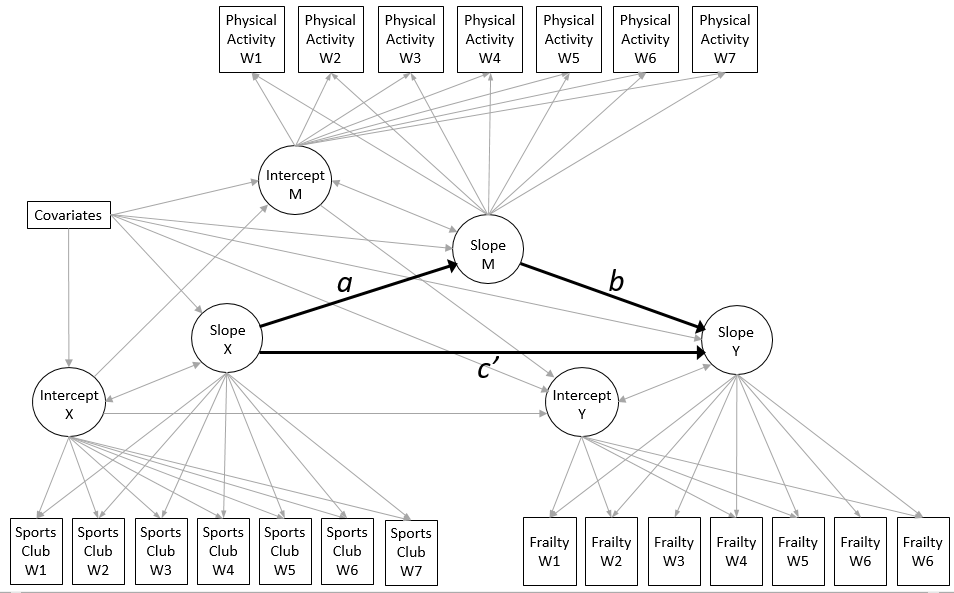


*Results*

The mediational LGCM (Figure A) had very good model fit measured by a range of model fit criteria (RMSEA = 0.009, [90%CI = 0.008, 0.010]; CFI=0.995; TLI=0.994). We found no evidence of significant average change in the dependant variables over time, but found significant variance between individuals in sports club membership (Est = 0.020, [95%CI = 0.014,0.025]), physical activity (Est = 0.015, [95%CI = 0.011,0.018]) and frailty (Est = 0.001 95%CI = [-0.001,0.002]).

Gender was associated with increased probability of initial sports club membership (Est = 0.134, [95%CI = 0.073,0.196]), lower initial MVPA levels (Est = -0.052, [95%CI = -0.099,-0.004]) and higher initial frailty scores (Est = 0.013, [95%CI = 0.008,0.019]). Gender was not associated with change in sports club membership, MVPA or frailty over time. Older age was associated with a lower probability of initial sports club membership (Est = -0.013, [95%CI = -0.018,-0.009]) but not with change in sports club membership. Older age was associated with decreases in MVPA (Est = -0.004, [95%CI = -0.003,-0.005]) and increases in frailty over time (Est = 0.001, [95%CI = 0.000, 0.002]). Table A shows associations between all covariates and the LCGM parameters..

Before adjusting for MVPA, increases in propensity towards sports club membership were associated with decreased frailty scores over time (Est = -0.012, [95%CI = -0.022,-0.001]). Increases in sports club membership were associated with increases in regular MVPA (path *a,* Est = 0.258 [95%CI = 0.165,0.350]) and, in turn, increases in MVPA was associated with lower frailty scores (path *b,* Est = -0.068, [95%CI = -0.081,-0.054]). The product of path *a* and path *b* (i.e the ‘indirect effect’ of sports club membership on frailty, through physical activity) was statistically significant (Est = -0.164 [95% CI = -0.232, -0.096]).

|  | I (sports clubs) | | S (sports clubs) | | I (physical activity) | | S (physical activity) | | I (frailty) | | S (frailty) | |
| --- | --- | --- | --- | --- | --- | --- | --- | --- | --- | --- | --- | --- |
| Predictors | Est | 95%CI | Est | 95%CI | Est | 95%CI | Est | 95%CI | Est | 95%CI | Est | 95%CI |
| Men | Reference | | Reference | | Reference | | Reference | | Reference | | Reference | |
| Women | 0.141*** | (0.077,0.206) | 0.088 | (-0.042,0.218) | -0.066* | (-0.127,-0.006) | -0.044 | (-0.151,0.062) | 0.101*** | (0.060,0.142) | -0.018 | (-0.105, 0.069) |
| Age | -0.014*** | (-0.018,-0.009) | -0.007 | (-0.018,0.003) | -0.014*** | (-0.018,-0.010) | -0.028*** | (-0.036,-0.021) | 0.009*** | (0.005,0.012) | 0.022*** | (0.015, 0.030) |
| Managerial | Reference | | Reference | | Reference | | Reference | | Reference | | Reference | |
| Intermediate | -0.143** | (-0.232,-0.055) | -0.053 | (-0.218,0.112) | -0.079 | (-0.166,0.008) | 0.081 | (-0.066,0.227) | 0.092** | (0.031,0.154) | 0.056 | (-0.066, 0.179) |
| Routine and manual | -0.283*** | (-0.368,-0.199) | -0.121 | (-0.293,0.050) | -0.181*** | (-0.264,-0.099) | 0.007 | (-0.137,0.151) | 0.231*** | (0.175,0.287) | 0.067 | (-0.048, 0.182) |
| Not living with a spouse/partner | Reference | | Reference | | Reference | | Reference | | Reference | | Reference | |
| Living with a spouse/partner | 0.081* | (0.006,0.155) | 0.053 | (-0.096,0.203) | 0.198*** | (0.131,0.264) | 0.000 | (-0.119,0.120) | -0.208*** | (-0.251,-0.165) | 0.105* | (0.012, 0.199) |
| White ethnicity | Reference | | Reference | | Reference | | Reference | | Reference | | Reference | |
| Non-white ethnicity | -0.146 | (-0.396,0.104) | -0.003 | (-0.515,0.509) | -0.284** | (-0.490,-0.078) | -0.233 | (-0.638,0.172) | 0.267*** | (0.146,0.389) | 0.159 | (-0.152, 0.470) |
| No qualifications | Reference | | Reference | | Reference | | Reference | | Reference | | Reference | |
| Intermediate qualifications | 0.354*** | (0.263,0.445) | 0.036 | (-0.153,0.225) | 0.310*** | (0.222,0.398) | -0.051 | (-0.203,0.274) | -0.233*** | (-0.293,-0.173) | 0.097 | (-0.024, 0.218) |
| Higher education or above | 0.475*** | (0.367,0.583) | 0.195 | (-0.023,0.413) | 0.518*** | (0.405,0.630) | 0.079 | (-0.116,0.120) | -0.343*** | (-0.422,-0.265) | 0.098 | (-0.068, 0.264) |
| Non-smoker | Reference | | Reference | | Reference | | Reference | | Reference | | Reference | |
| Smoker | -0.488*** | (-0.580,-0.395) | 0.025 | (-0.188,0.239) | -0.211*** | (-0.287,-0.135) | -0.299*** | (-0.438,-0.161) | 0.152*** | (0.101,0.202) | 0.112 | (-0.001, 0.225) |
| Not in paid employment | Reference | | Reference | | Reference | | Reference | | Reference | | Reference | |
| Currently in paid employment | 0.022 | (-0.057,0.101) | 0.034 | (-0.112,0.180) | .0252*** | (0.175,0.329) | 0.142* | (0.017,0.266) | -0.598*** | (-0.650,-0.547) | 0.313*** | (0.207, 0.419) |
| I (sports clubs) |  | | -0.223*** | (-0.346,-0.099) | 0.413*** | (0.379,0.448) |  |  | -0.272*** | (-0.306,-0.238) |  |  |
| S (sports clubs) |  |  |  |  |  |  | 0.269*** | (0.174,0.364) |  |  | 0.054 | (-0.056,0.163) |
| I (physical) activity |  |  |  |  |  |  | -0.371*** | (-0.446,-0.296) | -0.578*** | (-0.607,-0.550) |  |  |
| S (physical activity) |  |  |  |  |  |  |  |  |  |  | -0.610*** | (-0.711,-0.510) |
| I (frailty) |  |  |  |  |  |  |  |  |  |  | -0.420*** | (-0.470,-0.369) |

***Table A. Associations between covariates and LCGM model parameter.***

|  | Est | 95%CI |
| --- | --- | --- |
| Path *a* (x🡪m) | 0.269*** | (0.174,0.364) |
| Path *b* (m🡪y) | -0.610*** | (-0.711,-0.510) |
| *a***b* (indirect ‘effect’) | -0.164*** | (-0.232-0.096) |
| Path c’ (direct ‘effect’) | 0.054 | (-0.056, 0.163) |
| Path *c* (total ‘effect’) | -0.111* | (-0.210-0.011) |

***Table B. Direct, indirect and total associations between sports club membership, physical activity and frailty in the mediational LCGM.***

**References**

[1] E. Cerin, "Ways of unraveling how and why physical activity influences mental health through statistical mediation analyses," *Mental Health and Physical Activity,* vol. 3, no. 2, pp. 51-60, 2010.

[2] S. T. Khoo, "Assessing program effects in the presence of treatment—baseline interactions: A latent curve approach," *Psychological Methods,* vol. 6, no. 3, p. 234, 2001.
